# Central Proliferation and Neurogenesis Is Impaired in Type 2 Diabetes and Prediabetes Animal Models

**DOI:** 10.1371/journal.pone.0089229

**Published:** 2014-02-20

**Authors:** Juan Jose Ramos-Rodriguez, Sara Molina-Gil, Oscar Ortiz-Barajas, Margarita Jimenez-Palomares, German Perdomo, Irene Cozar-Castellano, Alfonso Maria Lechuga-Sancho, Monica Garcia-Alloza

**Affiliations:** 1 Division of Physiology, Dpt. Biomedicine, Biotechnology and Public Health, Institute of Biomolecules (INBIO), School of Medicine, University of Cadiz, Cadiz, Spain; 2 Institute de Biology and Molecular Genetics, University of Valladolid-CSIC, Valladolid, Spain; 3 Dpt. of Inorganic and Organic Chemistry, and Biochemistry, University of Castilla La Mancha, Toledo, Spain; 4 Department of Pediatrics, School of Medicine, University of Cadiz, Cadiz, Spain; University of Lancaster, United Kingdom

## Abstract

Type 2 diabetes (T2D) is an important risk factor to suffer dementia, including Alzheimer’s disease (AD), and some neuropathological features observed in dementia could be mediated by T2D metabolic alterations. Since brain atrophy and impaired neurogenesis have been observed both T2D and AD we analyzed central nervous system (CNS) morphological alterations in the db/db mice (leptin receptor KO mice), as a model of long-term insulin resistance and T2D, and in C57Bl6 mice fed with high fat diet (HFD), as a model of diet induced insulin resistance and prediabetes. Db/db mice showed an age-dependent cortical and hippocampal atrophy, whereas in HFD mice cortex and hippocampus were preserved. We also detected increased neurogenesis and cell proliferation rates in young db/db mice when compared with control littermates. Our study shows that metabolic parameters serve as predictors of both atrophy and altered proliferation and neurogenesis in the CNS. Moreover in the cortex, atrophy, cell proliferation and neurogenesis were significantly correlated. Our data suggest that T2D may underline some of the pathological features observed in the dementia process. They also support that blood glucose control in elderly patients could help to slow down dementia evolution and maybe, improve its prognosis.

## Introduction

Increasing life expectancy is secondarily rising the incidence of pathologies associated with aging, among which dementia processes and type 2 diabetes (T2D) are of special relevance, due to their high prevalence as well as the personal and social burden. T2D complications include nephropathy, cardiovascular disease, neuropathies, or cognitive decline and dementia [1], [2], [3]. Moreover previous studies have pointed to the fact that T2D and dementia, concretely Alzheimer’s disease (AD), could be related and share common underlying mechanisms [4], [5], [6], and in order to further explore this relationship recent studies have attempted to reproduce both pathologies in a single animal model [7], [8], [9]. Pathological features of AD include senile plaques, neurofibrillary tangles and final neuronal loss and central atrophy. Hyperinsulinemia and impaired glucose tolerance could enhance the progression of neurodegeneration, synaptic loss and brain atrophy, responsible for the cognitive decline observed in dementia [2]. On the other hand, although still controversial (for review see [10]), adult neurogenesis also seems to be altered in AD models [11] and AD patients [12]. Adult neurogenesis occurs in two mayor areas in the brain: the subventricular zone (SVZ) of the lateral ventricles and the dentate gyrus [13]. Although to a lesser extent, other areas, including the cortex [14], have shown some neuroregenerative capacity. Adult neurogenesis may be classified in stages including proliferation of neuronal progenitor cells, fate specification and maturation, and selective survival of newborn neurons [10]. It has been described that T2D can also interfere with adult neurogenesis in animal models [13], [15], playing a role in long-term potentiation and cognition [16]. Moreover, antidiabetic drugs such as GLP-1 analogues (liraglutide and exendin-4), enhance long-term potentiation [17], [18], as the basis for memory formation. Taking into account these considerations it seems feasible that central atrophy and impaired adult neurogenesis could be related, and underline the implication of T2D in the dementia process.

Db/db mice, as a T2D animal model, have been widely used in the literature however, central characterization remains limited and whether aging influences brain atrophy and central cell proliferation remains uncertain. On the other hand whether severe diabetes or just the prediabetes state of hyperinsulinemia can induce CNS alterations remains controversial, and observations seem to depend on the experimental approach to induce hyperinsulinemia, and the specific tests performed afterwards [19], [20], [21]. Therefore the aim of this study was to explore whether dementia-related alterations in the central nervous system (CNS) such as cortical and hippocampal atrophy, cellular proliferation and neurogenesis in relevant regions, including SVZ, hippocampus and cortex, could be affected in: 1) young db/db mice (4 weeks old), when glucose levels are still controlled by increased insulin levels, 2) adult db/db mice (14 weeks old), when T2D has already clinically started, and 3) older db/db mice (26 weeks old), when T2D has evolved. We also included in our study 26 weeks old C57Bl6 mice, in a prediabetes state, induced by long-term high fat diet (HFD). We observed an age-dependent atrophy process that affects the cortex preferentially in db/db mice, and increased central cell proliferation and neurogenesis, especially relevant in young animals. Interestingly, metabolic parameters resulted reliable predictors for cortical atrophy as well as proliferation and neurogenesis. Moreover, cortical cell proliferation and neurogenesis also seem to be associated with observed cortical atrophy.

## Materials and Methods

### 1. Animals

We used the db/db mouse as a model of obesity and type 2 diabetes in this study. The introduction of an *Rsa*I site by the *Leprdb* mutation in the leptin receptor gene was detected by 126 PCR as previously described [9]. C57BL/KsJ heterozygous db/+ mice were purchased from Harlan Laboratories (Boxmeer, The112 Netherlands). WT, db/db and db/+ mice were generated from crosses between heterozygous db/+ mice. These animals received regular chow and were aged up to 4, 14 and 26 weeks of age and 5–6 animals/group were included in the studies. Since heterozygous (db/+) mice do not show specific phenotype [9], WT and db/+ mice were included in the control group.

We also included a hyperinsulinemic prediabetes animal model. We used age-matched C57Bl6 mice (Harlam, Boxmeer. Holanda) fed with high fat diet (HFD) (60% kcal from fat, OpenSource, New Brunswick, NJ, USA) for 18 weeks. HFD feeding started when mice were 8 weeks old and ended at the age of 26 weeks, as the oldest db/db group. Control mice for this group, were age-matched C57Bl6 mice receiving regular diet from our animal facility: SAFE A04 (Augy, France).

All mice were housed in a temperature controlled environment, with a 12 hour dark/light cycle and all experimental procedures were approved by the Animal Care and Use Committee of the University of Cadiz, in accordance with the Guidelines for Care and Use of experimental animals (European Commission Directive 86/609/CEE and Spanish Royal Decree 1201/2005).

### 2. Metabolic Determinations

Body weight, postprandial blood glucose and insulin levels were determined immediately before sacrifice at all study points (4, 14 and 26 weeks) in db/db mice, as previously described [9]. Body weight, postprandial glucose and insulin levels were also determined in C57Bl6 mice after 18 weeks on HFD (26 weeks of age). Briefly, blood glucose levels were measured from nicked tails using the glucometer Optium Xceed (Abbott, United Kingdom). Blood for plasma insulin determination was collected from the tail vein into capillary tubes precoated with potassium-EDTA (Sarstedt, Nümbrecht, Germany). Plasma insulin levels were measured using ultrasensitive mouse enzyme-linked immunosorbent assay (ALPCO Diagnostics, Salem, NH).

### 3. Brain Histomorphology and Cresyl Violet Staining

Animals were sacrificed at 4, 14 and 26 weeks of age with chloral hydrate (60 mg/Kg i.p.). Brains were harvested and weighted at selected times. Hemispheres were fixed in 4% PFA for 2 weeks before 30 µm coronal brain sections were cut. For cresyl violet staining, sections were selected at 1.5, 0.5, −0.5, −1.5, −2.5 and −3.5 mm from Bregma [22], as previously described [23], in order to cover the cortex and hippocampus. Briefly, sections were mounted and dehydrated in 70% ethanol for 15 minutes before incubation in cresyl violet (Sigma, St. Louis, MO, USA) solution 0.5% w/v for 10 minutes. Sections were washed and fixed in 0.25% acetic acid in ethanol for 7 minutes and subsequent 100% ethanol and xylene for 2 minutes. Sections were mounted with DPX (Sigma, St. Louis, MO, USA) and images were acquired using an optical Olympus Bx60 microscope (Japan) with an attached Olympus DP71 camera and Cell F software (Olympus, Hamburg, Germany). Brain morphology was analyzed in 6 cresyl violet stained sections selected 1 mm apart (from 1.5 to −3.5 mm from Bregma) [22] as previously described [23]. Cortical thickness was measured in frontal, parietal and temporal cortical sections, and hippocampal thickness was measured at the dental gyrus, as well as in CA1 and CA3, using Adobe Photoshop Elements and Image J softwares.

### 4. 5-Bromo-3-deoxyuridin and Doublecortin Immuno Histochemistry

Contiguous sections to those used for cresyl violet staining (from 1.5 to −3.5 from Bregma) [23] were used to analyze 5-Bromo-3-deoxyuridine (BrdU) and doublecortin (DCX) immunohistochemistry in the cortex and hippocampus, where all individual cells in whole sections were quantified. We also selected coronal sections including subventricular zone (SVZ) in order to quantify BrdU and DCX in this neurogenic niche (0.5, and 0.0 mm from Bregma). Briefly sections were washed in 0.1 M PBS and antigen retrieval procedure included citrate buffer and formamide (1∶1) for 2 h at 65°C. Sections were washed in citrate buffer and further incubated in 2N hydrochloric acid for 30 min at 37°C. Sections were washed in 25 mM borate buffer (pH = 8.4) and rinsed off in 0.1 M PBS. Blocking was carried in 0.1% triton-X in 2.5% BSA (Sigma, Or, USA) and 0.25% sodium azide for 1 h at room temperature. Primary antibodies were diluted in blocking solution: Monoclonal Mouse anti-BrdU 1∶100 (Dako, Barcelona, Spain) and polyclonal IgG Goat anti-DCX 1∶400 (SantaCruz Biotechnology, Santa Cruz, CA, USA) at 4°C overnight. After washing, sections were incubated with secondary antibodies Alexa Fluor 594 and Alexa Fluor 488 1: 100 (Invitrogen, Carlsbad, CA, USA). Images were obtained in a fluorescent microscope (Olympus Bx60, Japan) with a camera (Olympus DP71). SVZ is extremely rich in BrdU and DCX positive cells and in order to analyze the complete border of the ventricle, contiguous images 20 µm from the ventricle lumen, were acquired and merged using Photoshop Elements software. We quantified the number of individual BrdU-positve cells in the SVZ using Image J. Since the number of DCX-positive cells is very high in the SVZ, delimiting individual cell cytoplasms could not be done in a reliable manner. Therefore DCX burden (percentage of area covered by DCX-positive cells) was quantified in the SVZ, using Image J free software. In the cortex and hippocampus the number of individual DCX- and BrdU-positive cells was quantified using Image J software.

### 5. Statistical Analysis and Correlation Studies

Metabolic assessment, histomorphology studies and immunohistochemistry quantification were analyzed by one-way ANOVA for independent samples followed by Tuckey b test or Tamhane test as required. Spearman rank’s correlations were used to perform correlation studies between BrdU and DCX as well as to explore correlations between metabolic, histomorphological and cell proliferation-neurogenesis markers.

## Results

### 1. Metabolic Parameters and T2D Progression in db/db and HFD-treated Mice

In our hands, db/db mice developed hyperinsulinemia and T2D with aging, as previously described [23]. At 4 weeks of age db/db mice showed an increase in body weight, although differences did not reach statistical significance. However by 14 weeks of age a significant increase in body weight was detected in db/db mice and a similar profile was observed at 26 weeks of age (table 1). Wild-type mice fed with a HFD also showed a progressive increase in body weight (data not shown) and, by the age of 26 weeks (after receiving HFD for 18 weeks) values were comparable to those observed in 26-week old db/db mice (table 1). A similar profile was observed with glucose levels, and whereas by 4 weeks of age no differences were detected in db/db mice by 14 weeks T2D was established in db/db mice, with glucose levels above 300 mg/dl in plasma, reproducing our previous studies [23], and this effect was also observed at 26 weeks of age. On the other hand although glucose levels were higher in HFD fed mice than in control mice, these were still far from reaching 300 mg/dl (table 1). Our data are in accordance with previous studies and insulin levels were also in the same range of those observed before [23]. A slight increase in insulin levels was observed in db/db mice at 4 weeks of age, although these data did not reach statistical significance when compared with the rest of the groups (table 1). By 14 weeks although hyperinsulinemic, high insulin levels were no longer able to compensate for chronically increased glucose levels, indicating insulin resistance. At 26 weeks of age, metabolic parameters in db/db mice were similar to those measured at 14 weeks (table 1). Wild-type mice fed with a HFD also showed a progressive increase in insulin levels and, by the age of 26 weeks (after receiving HFD for 18 weeks) this effort was capable to control glucose levels, when compared to RD controls. Therefore, we considered HFD-treated mice as a model for prediabetic hyperinsulinemia, as previously described [20], [21].

**Table 1:** Body weight, glucose and insulin levels in 4, 14 and 26 weeks db/db and Control mice as well as in 26 weeks C57Bl6 mice after 18 weeks fed with HFD.

	Age	Body weitght (g)	Glucose (mg/dl)	Insulin (ng/ml)
**Control 4 weeks**	**4 weeks**	7.58±0.77	74.60±14.15	0.36±0.01
**db/db 4 weeks**		8.76±0.71	86.80±9.91	1.20±0.73
**Control 14 weeks**	**14 weeks**	25.48±2.38	124.60±4.53	0.46±0.08
**db/db 14 weeks**		43.60±2.16**	550.20±49.80**	4.74±1.06**
**Control 26 weeks**	**26 weeks**	25.48±2.38††	102.80±8.36	0.39±0.04
**db/db 26 weeks**		40.39±2.78**	484.40±34.31**	4.87±1.29**
**RD**		29.60±1.41††	124.99±9.72	0.60±0.07
**HFD**		44.53±1.74**	142.70±7.17	9.47±2.30††

Metabolic parameters including body weight, glucose and insulin levels were determined at the end of the experiments. Differences were detected by one-way ANOVA followed by Tuckey b or Tamhane tests as required. When we compared all groups under study we observed a significant increase in body weight as age progressed, and even higher body weights were observed in overweight mice (db/db 14 weeks old mice, db/db 26 weeks old mice and HFD treated mice), when compared to the rest of the groups [F_(7,35)_ = 63.03, **p<0.01 vs. Control 14 weeks, Control 26 weeks, RD and 4 weeks old mice (db/db and Control), ††<0.01 vs. Control and db/db 4 weeks]. Glucose levels were significantly higher in db/db mice both at 14 and 26 weeks of age when compared with the rest of the groups [F_(7,35)_ = 74.36, **p<0.01 vs. Control 14 weeks, Control 26 weeks, RD, HFD and 4 weeks old mice (db/db and Control)]. Increased insulin levels were observed in db/db 4 weeks mice, although these differences did not reach statistical significance. Significant hyperinsulinemia was observed in db/db 14 weeks and db/db 26 weeks whereas statistically higher levels were observed in HFD treated mice [F_(7,35)_ = 11.498, **p<0.01 vs. Control 14 weeks, Control 26 weeks, RD and 4 weeks old mice (db/db and Control), ††p<0.01 vs. rest of the groups].

### 2. Brain Histomorphology in db/db and HFD-treated Mice

**Figure 1:**
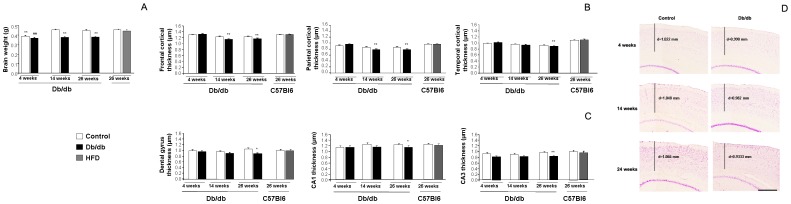
Brain atrophy in db/db mice. **A**) Brain weight was significantly reduced is db/db mice as disease progressed. At 4 weeks of age brains were significantly smaller in Control and db/db mice, when compared with adult brains. As T2D worsened a significant reduction in brain weight was observed in 14 and 26 weeks of age db/db mice, whereas no effect of prediabetes was observed in HFD treated mice [F_(7,31)_ = 23.70, **p<0.01 vs. Control 14 and 26 weeks, C57 RD and C57 HFD]. **B**) Cortical thickness affected as disease progressed and specific cortical areas were significantly reduced. Frontal cortex was thinner in db/db mice by 14 weeks of age and this effect was worsened by 26 weeks of age [F_(7,337)_ = 11.683, **p<0.01 vs. Control 14 weeks, Control 26 weeks, Control and db/db 4 weeks, RD and HFD treated mice]. A similar profile was observed in the parietal cortex and a progressive thinning effect was observed in db/db mice [F_(7,161)_ = .34, **p<0.01 vs. Control 14 weeks, Control 26 weeks, Control and db/db 4 weeks, RD and HFD treated mice]. Temporal cortex was only affected in 26 weeks db/db mice when compared with the rest of the groups [F_(7,250)_ = 114.232, **p<0.01 vs. Control and db/db 4 weeks, RD and HFD treated mice]. **C**) A similar profile was observed in the hippocampus, although thinning effect was detected at later stages. A significant reduction in dental gyrus thickness was only detectable in db/db mice at 26 weeks of age [F(7,118) = 1.11, *p = 0.47 vs. Control 26 weeks]. Hippocampal CA2 thickness was also significantly reduced in db/db 26 weeks [F(7,116) = 2.313, *p = 0.30 vs. Control 26 weeks]. Although a reduction in CA3 thicknes was observed in db/db mice at 14 weeks of age, differences only reached statistical significance in db/db 26 weeks mice [F(7,115) = 2.970, **p = 0.007 vs. RD and HFD treated mice]. **D**) Illustrative example of cortical thinning in db/db and control mice at 4, 14 and 26 weeks of age. Scale bar: 500 µm.

Previous studies have shown a significant age-dependent brain shrinkage in db/db mice [23], in a similar way to that observed in AD patients. We also observed that brain weight was significantly reduced as disease progressed and we detected a significant ageXgenotype effect: F_(1,2)_ = 13.097, **p<0.01. When we compared all groups under study we observed that 4 weeks both Control and db/db mice had significantly smaller brains, probably due to the fact that by 4 weeks mice have not reached adulthood (Figure 1A) and the fact that no differences were observed at this age between db/db and control mice, support the idea that brain atrophy has not started at this age. However by 14 weeks of age brain weight was significantly reduced in 14 weeks old db/db mice and this effect was worsened by 26 weeks of age, as disease progressed. On the other hand brain weight in HFD-treated mice seemed to be preserved. In order to detect specific implication of relevant learning and memory areas, we measured cortical and hippocampal thickness by cresyl violet staining. An overall cortical and hippocampal size reduction has been previously observed [23], however specific reduction in cortical thickness seems to be a more widely used measurement in human studies [24], [25] and to our knowledge selective implication of cortical and hippocampal regions has not been assessed. We observed an age dependent reduction of cortical thickness and a significant ageXgenotype effect in all cortical measurements performed in the db/db colony (frontal cortex: F_(1,2)_ = 18.975, **p<0.01; temporal cortex F_(1,2)_ = 10.707, **p<0.01; parietal cortex: F_(1,2)_ = 14.591, **p<0.01) in db/db mice. While cortical thickness was preserved at 4 weeks of age, when animals are still normoglycemic, at older ages (14 and 26 weeks of age) we observed that frontal and temporal cortex were significantly thinner, whereas temporal areas were preserved (figure 1B and D). On the other hand HFD-treated mice showed no significant cortical thinning in any of the assessed areas (figure 1B).

We also observed an age dependent hippocampal thinning in db/db mice, although hippocampus seems to be preserved at early stages of the disease. A significant thinning effect is only detected at 26 weeks of age, when T2D is fully established and has evolved, affecting the whole hippocampal area. On the other hand, hippocampus seems to be preserved in db/db mice at 4 and 14 weeks of age (figure 1C). Following these observations, prediabetic HFD-treated mice show no statistical differences in hippocampal thickness (figure 1C).

### 3. Cellular Proliferation and Neurogenesis in db/db and HFD-treated Mice

**Figure 2:**
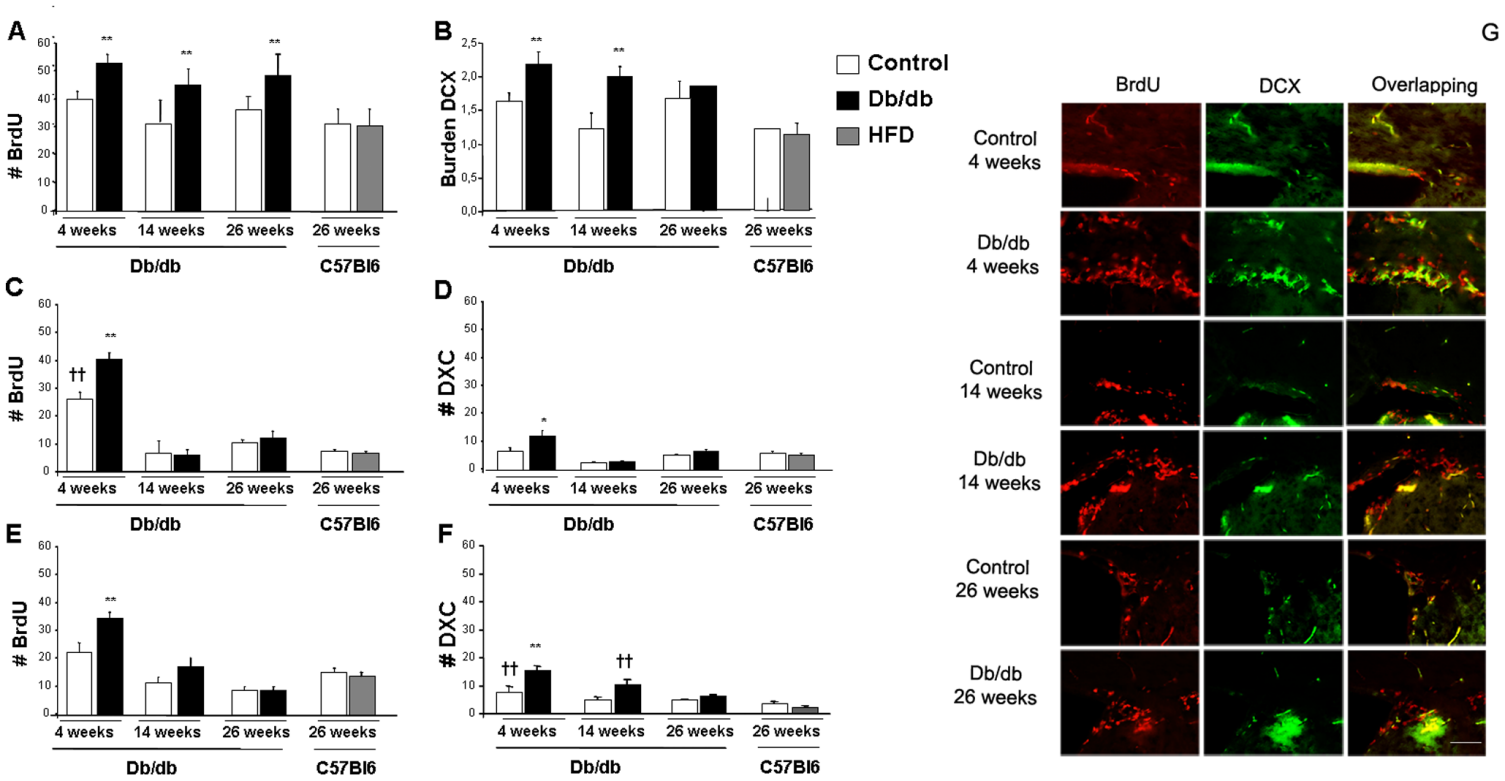
Central proliferation and neurogenesis is altered in db/db mice. Significant alterations were observed in the SVZ, cortex and hippocampus of db/db in an age dependent manner. Differences detected by one way ANOVA followed by Tuckey b or Tamhane tests as required. **A**) Db/db mice showed increase in the number of BrdU-positive cells in the SVZ, more evident at early ages (4 weeks), although at 14 and 26 weeks of age the increase of BrdU-possitive cells is still present. No effect was observed in HFD-treated mice [F_(7,68)_ = 5.624, **p<0.01 vs. Control, RD and HFD-treated mice]. **B**) DCX burden (% area covered by DCX staining) was also significantly increased in db/db mice at early stages (4 and 14 weeks) whereas no statistical differences were detected at 26 weeks or in HFD-treated mice [F_(7,73)_ = 1.761, **p = 0.03 vs. Control, RD and HFD-treated mice]. **C**) We observed an age dependent effect of the proliferation process in the cortex of our mice. A significantly higher number of BrdU-possitive cells was observed in Control 4 weeks mice and this effect was even higher in case of db/db mice, whereas no other effect was observed in the rest of the groups under study [F_(7,202)_ = 81.08, **p<0.01 vs. rest of the groups, ††p<0.01 vs. 14 weeks and 26 weeks old (Control and db/db) as well as RD and HFD-treated mice]. **D**) A similar profile was observed in the number of DCX-positive cells in the cortex, where we detected significant increase in DCX labelling in 4 weeks old db/db mice when compared with the rest of the groups [F_(7,196)_ = 12.232, **p<0.01 vs. rest of the groups]. **E**) In the hippocampus we observed a similar profile to that measured in the cortex. We detected an age dependent effect in the number of BrdU-positive cells in the hippocapmus, and differences reached statistical significance in the case of 4 weeks old db/db mice [F_(7,118)_ = 111.058, **p<0.01 vs. Control, 14 and 26 weeks db/db mice and RD and HFD-treated mice]. **F**) The number of DCX-positive cells was also reduced with aging in the hippocampus, mostly in db/db mice [F_(7,117)_ = 129.94, **p<0.01 vs. ret of the groups, †† vs. Control 14 and 26 weeks, db/db 26 weeks and RD and HFD-treated mice]. **G**) Representative image of BrdU (red) and DCX (green) staining in SVZ from control and db/db mice at 4, 14 and 26 weeks of age. Scale bar 100 µm.

We assessed central proliferation and neurogenesis in db/db and HFD-treated mice. For this purpose SVZ, a major neurogenic site in the adult brain, was analyzed and we observed that db/db mice presented significantly higher number of BrdU-positive cells when compared to control mice (Figure 2A and figure 2G). Although a slight reduction in the number of BrdU-positive cells was observed with aging, we did not detect a significant ageXgenotype effect in the db/db colony [F_(2,1)_ = 1.123, p = 0.333]. This was supported when long-term treatment with HFD mice where included in the study. No differences in cell proliferation in SVZ from 26 weeks old C57Bl6 mice on HFD were observed, and quantification values were similar to those observed in control mice. However a significant increase in BrdU-possitive cells could be detected in db/db mice when compared with control mice (figure 2A and 2B). Double immunostaining with DCX also showed a similar profile in db/db mice, although significant differences were only observed in young db/db mice (4 and 14 weeks of age), supporting that neurogenic process is favoured at early stages of the disease, whereas at later time points (26 weeks of age) differences were not statistically significant (figure 2B and 2G). We did not detect a significant ageXgenotype effect in the number of DCX-positive cells in the db/db colony [F_(2,1)_ = 2.017, p = 0.143]. Again long-term HFD-treated mice were not significantly different from control mice at 26 weeks of age. In order to explore whether cellular proliferation and neurogenesis is affected in other relevant areas for learning and memory we analyzed the hippocampal dental gyrus and the cortex. We observed that proliferation was favoured at young stages (4 weeks old) and that db/db mice had significantly higher number of BrdU-positive cells in the cortex, whereas at later stages (14 and 26 weeks of age) the amount of BrdU-positive cells was limited, and no differences were detected between db/db and control mice (figure 2C). Once more HFD-treated mice showed no significant changes, suggesting that long-trem HFD has no relevant effect on hippocampal cell proliferation (figure 2C). In the cortex we detected a significant ageXgenotype effect in the proliferation process in the db/db colony [F_(2,1)_ = 14.538, **p<0.01]. A similar profile was observed when neurons were specifically counted, and only at 4 weeks of age was neurogenesis favoured in db/db mice (figure 2D). Although the number of DCX-positive cells was reduced with aging we did not detect a significant ageXgenotype effect in the number of neurons generated in the db/db colony [F_(2,1)_ = 1.469, p = 0.234], showing a similar evolution for control and db/db mice. HFD-treated mice showed no differences when neurogenesis was measured in the cortex (figure 2C and 2D). Dental gyrus was also analyzed and a similar profile to that observed in the SVZ was detected, and only at early stages cell proliferation and neurogenesis was increased in db/db mice (figure 2E and 2F). Both in the cell proliferation and neurogenesis processes we detected a significant agexgenotype effect in the db/db colony ([F_(2,1)_ = 0.315, *p = 0.044] and [F_(2,1)_ = 13.322, *p = 0.041] respectively). No differences were detected in HFD-treated mice at 26 weeks of age when compared with the rest of the groups (figure 2E and 2F).

### 4. Metabolic and Morphological Correlations

**Figure 3:**
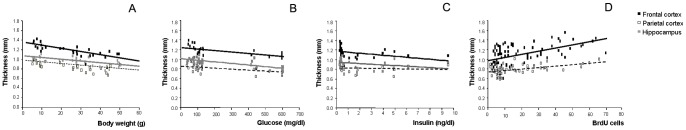
Metabolic parameters are good predictors of cortical and hippocampal atrophy and neurogenesis abnormalities in db/db mice. Significant negative correlations were detected between metabolic parameters (body weight, glucose and insulin levels) and cortical thickness when all animals under study were analyzed. Data are representative of 30 mice and significant correlations (**p<0.01 and *p<0.05) were detected by Spearman’s rank correlations as follow: **A**) Body weight/frontal cortex: −0.813**, parietal cortex: −0.696**, hippocampus: −0.539**; **B**) Glucose levels/frontal cortex: −0.686**; parietal cortex: −0.506**, hippocampus: −0.597*; **C**) Insulin levels/frontal cortex: −0.657**, parietal cortex: −0.472**, hippocampus: −0.510**; **D**) BrdU/frontal cortex: 0.40**, parietal cortex: 0.517**.

In order to determine whether central nervous system alterations in cell proliferation and neurogenesis processes could be predicted by metabolic parameters we performed Spearman’s rank correlations and we detected an overall negative correlation between body weight, glucose and insulin levels, with the number of BrdU-positive cells in the cortex and hippocampus of all db/db mice (table 2). On the other hand we observed that the metabolic parameters were also correlated with the number of DCX-positive cells, in cortex and even stronger correlations were observed in the hippocampus (table 2). These data suggest that basic metabolic parameters may serve as predictors of cell proliferation and neurogenesis in relevant areas for learning and memory, such as cortex and hippocampus. We also detected that body weight, glucose and insulin levels were also negatively correlated with frontal and parietal cortex thickness (figure 3A and B). A similar profile was observed in the hippocampus (figure 3C). Our observations support that metabolic parameters may partially predict the cortical atrophy observed in T2D. Further analysis also revealed that in the cortex, where atrophy is earlier detected and more severe, frontal and parietal cortex thickness is correlated with the number of BrdU-positive cells, suggesting that proliferation capacity is reduced as cortical thinning increases and the disease progresses in db/db mice (figure 3D).

**Table 2:** Metabolic parameters correlations with the number of BrdU- and DCX-positive cells in the cortex and hippocampus, and cortical and hippocampal thickness of db/db mice.

		Body weight (g)	Glucose (mg/dl)	Insulin (ng/ml)
**Cortex**	# BrdU/section	−0.696**	−0.633	−0.485**
	# DCX/section	0.478**	0.400*	0.538**
**Hippocampus**	# BrdU/section	−0.472*	−0.401*	−0.207
	# DCX/section	0.677*	0.554**	0.554**

Body weight, glucose and insulin may serve as predictors of the number of BrdU and DCX positive cells in the cortex and the hippocampus of db/db mice (4, 14 and 26 weeks of age). Significant negative correlations were also detected between metabolic parameters and cortical thickness. Data are representative of 28–30 mice and significant correlations were detected by Spearman’s rank correlations are presented (**p<0.01 and *p<0.05).

## Discussion

The close relationship between T2D and dementia is becoming a hot topic in recent years and both epidemiological studies [26], [27], [28], [29] and experiments using animal models [7], [8], [9], [30] have tried to understand the underlying mechanisms of this association. In order to further explore CNS alterations in T2D we have assessed neuropathological features associated with dementia in a classical model of T2D, as it is the db/db mice, as well as in a prediabetic model, induced by chronic HFD. Although the db/db mouse has been widely used in the literature as a T2D and metabolic syndrome animal model [31], [32] not many studies have focussed in CNS alterations and behavioural consequences [33], [34], [35]. In this sense relevant aspects associated with dementia, including region-specific atrophy and neurogenesis impairment have only been partially assessed. To our knowledge age-dependent studies, where the effect of T2D progression on specific brain areas atrophy, cell proliferation and neurogenesis have not been performed. In our hands db/db mice showed and age-dependent cortical and hippocampal atrophy, that affected frontal and temporal cortex at relatively early stages of the disease, whereas hippocampus was secondarily affected and significant thinning was observed only at later stages (26 weeks old) when the disease has evolved, supporting previous studies where cortical and hippocampal areas were assessed [35]. We detected that frontal and parietal cortex were early affected in db/db mice, and by 14 weeks of age, the cortex was significantly thinner, probably due to carbohydrate metabolism impairment. Although different species do not necessarily evolve in parallel after similar insult, our observations are in accordance with MRI studies where cortical volume and cortical thickness are reduced in T2D patients [24], [25]. This effect seems to be present also in the hippocampus, in association with cerebral small vessel disease [23], however detecting preferentially affected areas is complicated in one time imaging studies. Cortical atrophy has been associated with cognitive impairment in T2D patients [36] and this effect has also been observed in db/db mice [35]. We did not detect any alteration in cortical or hippocampal thickness on HFD-treated mice, and these data are in accordance with previous studies with C57Bl6 mice on HFD, reporting that hippocampal synaptic function and long-term potentiation are preserved in C57Bl6 mice on HFD for up to ten moths [37].

On the other hand, although peripheral cell proliferation, including liver or pancreas has been studied in db/db mice [38], [39] limited attention has been paid to the role of cell proliferation and neurogenesis in central nervous system of db/db, in spite of the fact that T2D has been closely related to dementia, and neurogenesis seems to be altered in the dementia process [11], [12]. Cell proliferation and neurogenesis in db/db mice was assessed in relevant neurogenic areas, such as SVZ and hippocampus, as well as in the cortex, due to its role in learning and memory, and to our knowledge this is the first approach to explore age related alterations in db/db mice. We detected that cell proliferation and neurogenesis was increased up to 14 weeks of age in db/db mice in the most predominant neurogenic areas (SVZ and hippocampus) whereas in the cortex, only in very young mice (4 weeks) we could detect increased cell proliferation. Although it has been described a reduction in cell proliferation and neurogenesis in hippocampus from db/db mice [40] our observations are in accordance with recent studies where increased hippocampal cell proliferation and neurogenesis has been observed in ∼11–12 weeks old db/db mice [15]. Moreover this effect seems to be increased by treatment with GLP-1 receptor agonists, used to treat T2D [15], [41]. We did not detect any of these effects in HFD-treated mice as previously shown [15]. This could be interpreted as if it was the chronic hyperglycaemia, rather than the compensating hyperinsulinemia, the mayor player of this effect, since HFD mice exhibit even higher insulin levels than db/db mice of the same age, and only slightly increased glycaemia. On the other hand it is also feasible that the use of a very severe model of T2D, as it is the db/db mice is obscuring any other considerations. We also need to consider that the length of the treatment and composition of HFD, 60% Kcal fat in our case, could be responsible for inducing only a prediabetic state, with controlled glycaemia as previously described [42]. And therefore we cannot exclude that HFD maintained for even longer periods could lead to more severe effects. In this sense previous studies have shown that metabolic alterations associated to HFD, or derived central complications, are not necessarily reproducible. Whereas Heyward et at [21] have reported severe cognitive impairment in HFD-treated mice, other studies with C57Bl/6 mice on HFD, for up to ten months, have also reported preserved hippocampal synaptic function and long-term potentiation, as well as maintained performance in the Morris water maze test [37]. Therefore it seems feasible that different protocols, including different diet composition, length of the chronic administration or age of the rodents at the commencement of the experiments [20], [21], [23], [43] may strongly determine the outcome of the experiments.

Cell proliferation seems to occur in response to a damage or insult, and it also seems to be impaired with aging [10], therefore it is feasible that db/db mice present some capacity of response at early stages whereas further impairment, as aging and disease progress could limit the ability of the CNS to continue regeneration, as observed in 26 weeks of age mice. It is also necessary to point out that since db/db mice are lacking the leptin receptor it is feasible that observed alterations are not purely due to the diabetic process. Insulin has been closely related with normal CNS performance and neurogenesis [44], [45], and leptin signalling also plays a role in regulating synaptic function and memory formation among others [46]. Following this idea previous studies have shown the neuroprotective effect of leptin after different insults such as apoptotic stimuli, tumor necrosis factor alpha, 6-hydroxydopamine [47], [48]. Whereas other central complications in db/db mice have been studied, including spontaneous bleeding, increased tau phosphorylation or learning and memory dysfunction, cell proliferation and neurogenesis has only been partially addressed, and it may play a relevant role in the close relationship described for T2D and AD [4], [28], [49]. As previously stated, we are aware of the fact that leptin signalling implications in multiple central functions may hamper our results, however our approach is supported by the relevance of the db/db mice as a T2D model and associated central complications. Moreover the fact that proliferation and neurogenesis processes, as wells as brain atrophy, are age dependent, support the idea that changes are due to the diabetic process, since leptin signalling alterations are present from the beginning in the db/db mice.

When we determined the implication of metabolic parameters on central nervous system alterations in db/db mice, we observed that metabolic determinations including glucose levels, insulin levels and body weight are good predictors of cortical atrophy as well as cortical and hippocampal cell proliferation, supporting the role of the diabetic process in observed central alterations. Of note, previous studies in patients have also shown significant associations between insulin levels in T2D patients and brain alterations, detected by MRI [50], as well as between cortical and metabolic disturbances [51]. Curiously whereas worse metabolic conditions correlate with lower rates of central cell proliferation, affected metabolism also predicts increased neurogenesis rates, suggesting that as the pathology progresses overall cellular production is impaired, while the system tries to compensate the generation of new neurons.

Altogether our results suggest that db/db mice reproduce relevant alterations observed in dementia, including cortical and hippocampal atrophy, as well as cell proliferation and neurogenesis impairment. Interestingly metabolic parameters can predict many of these alterations, therefore it could be possible that controlling metabolic parameters associated with T2D, could improve disease control and dementia prognosis.
